# Effects of mental health interventions given at youth-friendly health services and integrated youth services: a systematic review protocol

**DOI:** 10.1136/bmjopen-2024-095714

**Published:** 2025-09-21

**Authors:** Mikael Andersén, Petra Vikman Lostelius, Annika Bring, Lena Ring, Camilla Nystrand, Erik M G Olsson

**Affiliations:** 1 Department of Women’s and Children’s Health, Uppsala University, Uppsala, Sweden; 2 Region Uppsala, Primary Care and Health, Uppsala, Sweden; 3 School of Health, Care and Social Welfare, Mälardalen University, Västerås, Sweden; 4 Clinic for Pain Rehabilitation Västmanland, Region Västmanland, Västerås, Sweden; 5 Department of Learning, Informatics, Management and Ethics, Karolinska Institute, Stockholm, Sweden

**Keywords:** Adolescents, Systematic Review, MENTAL HEALTH, Health Services

## Abstract

**Abstract:**

**Introduction:**

Although poor mental health among young people has been increasing in the past decades, many young people are reluctant to use traditional mental healthcare. To cater to the needs of young people, various youth-friendly treatment options have been developed. These include the youth-friendly health service (YFHS) standards put forth by the WHO in 2012 and the integrated youth services (IYS) for mental health developed in certain countries globally. However, no synthesis of the effect of these services on youth mental health has been conducted. The aim of the proposed study is to conduct a systematic review of the effect of mental health treatments conducted within YFHS and IYS clinics. The primary research question is what effect mental health interventions given at ‘youth-friendly’ clinics for treating mental health, such as IYS and YFHS, have on the mental health and quality of life (QoL) of young people?

**Methods and analysis:**

A preliminary search for other reviews on the topic was conducted during the first half of 2024, after which a protocol of the present study was registered in PROSPERO. In May 2024, a search was carried out in the PubMed, PsycINFO, CINAHL and Web of Science databases, which gave references for 12 738 papers to be screened for inclusion in the review, and a follow-up search was carried out in April 2025, yielding a further 2182 references. For inclusion, studies must have participants between 12 and 25 years of age; interventions be given at clinics designed to be ‘youth-friendly’ or given at an IYS; control condition, if any, consisting of standard care or waiting list; outcomes must be mental health symptomology or QoL. To be included, studies must be published from 2012 and onwards. Screening of titles and abstracts in the initial search was carried out independently by two reviewers. Screening of studies found in the follow-up search and in the reference lists of included articles will be carried out in the same way. Data analysis of the initial search was conducted in the latter half of 2024, while final data analysis including the results from the follow-up search is ongoing. The Cochrane risk of bias assessment tools will be used to assess bias of included articles, and certainty of the evidence will be evaluated according to the GRADE methodology. A meta-analysis of the results will be performed if a sufficient amount of homogenous data is found; otherwise, a synthesis without meta-analysis will be conducted.

**Ethics and dissemination:**

The proposed review may form a valuable synthesis of the state of the art of treatment options catering to young people. Investigating the effectiveness of YFHS or IYS in treating young people’s mental health may inform future directions for development and research. The present study does not need ethical approval, since only previously published, ethically approved data are used in the current study. The findings of the study will be disseminated through submissions to peer-reviewed journals and international conferences, as well as disseminated within the Swedish YFHS community.

**PROSPERO registration details:**

ID nr CRD42024528687.

**STRENGTHS AND LIMITATIONS OF THIS STUDY**

- The proposed study employs a broad search strategy, which facilitates capturing a diverse number of youth-friendly or integrated mental health treatment options.
- Combining both youth-friendly health service and integrated youth services as well as any other ‘youth-friendly’ treatment modality in the same study provides the research community with an overview of available interventions and the evidence base for them.
- Limitations of the study include difficulties in capturing all forms of youth-friendly interventions, partially caused by a lack of consensus concerning terminology used in studies describing different youth-friendly treatment options, as well as possible difficulties generalising treatment effects due to heterogeneous treatment options.

## Introduction

 Research in the past decades has shown a trend of increased mental health problems among adolescents and young people.^1^,^4^ This trend has been seen for different types of mental health problems, such as depression^5^ and anxiety,^6 7^ but also stress and subclinical symptoms of mental problems.^8^ The WHO has found that suicide is the third leading cause of death among adolescents and that depression is a leading cause of loss of disability-adjusted life years among young people globally.^9^ Systematic reviews have also shown a further increase in mental health problems among young people during the COVID-19 pandemic,^10^ exacerbating the problem. Adolescence and young adulthood are an important phase in physical, personal and social development for young people,^11 12^ and studies show that poor mental health during adolescence is often the start of problems that continue throughout the lifespan.^13^ Poor mental health has in turn been shown to increase the risk of adverse outcomes later in life, such as lower educational attainment and unemployment.^14 15^ Given these findings, it is imperative that society and healthcare services can adequately and swiftly identify poor mental health in this age group and offer suitable support and treatment.

Although mental health problems are increasing, young people are often unwilling to engage in regular mental healthcare.^16 17^ Findings indicate that this is due to a number of factors, such as problems concerning accessibility and acceptability,^18^ concerns around the stigma of poor mental health^19^ and the need for privacy and integrity which also includes confidentiality towards adult caregivers.^20 21^ Different solutions to adapting health services to the needs of adolescents and young people have been proposed. The WHO published guidelines in 2012^16^ for youth-friendly health services (YFHS), sometimes referred to as adolescent-friendly health services, in order to cater to the needs of young people and empower them to seek help. The guidelines have been formulated as eight Global Standards with a total of 79 measurement criteria,^17^ which can be summarised as YFHS being accessible, acceptable, equal, appropriate and effective.^22^ These guidelines may be applied to all forms of health services,^16^ and many countries have established networks of YFHS clinics predominantly for sexual and reproductive health as well as mental health.^16 23 24^ However, some countries still lack adequate YFHS for mental health needs,^23^ and studies have questioned whether YFHSs targeted at mental health may require different guidelines compared with other kinds of YFHSs.^25^

Another framework for catering to the specific mental healthcare needs of young people is the integrated youth services (IYS) for mental health.^26^ IYS have been established in certain countries globally, for example, in Australia,^26^ Canada^27^ and Ireland.^28^ These clinics share many of the same elements that form the basis for YFHS, such as focusing on availability and on the services being acceptable for young people, and have shown promising results in attracting young people.^26 29^ IYS, in literature often called ‘one-stop shops’ for mental health, are sometimes referred to in the literature as ‘youth-friendly’ or ‘youth-centred’,^26^ but IYS do not specifically follow YFHS guidelines and place explicit focus on integrating different forms of care, including social services and peer support, within one service.^26 27^ For this reason, although they share many similarities, IYS and YFHS should not be conflated.

There is a knowledge gap concerning the effectiveness of both IYS and YFHSs in treating mental health problems. Although evaluations of individual clinics and services exist, to our knowledge, no knowledge synthesis has yet provided an overview of the field as a whole. No systematic review has described the effects on mental health outcomes of treatments provided at either YFHS or IYS, entailing that no general conclusions concerning the effect of treatments in these services can be drawn. A review of treatment outcomes at these clinics, which are intended specifically to cater to young people, who as a group are reluctant to seek other care, could form an important basis for further development of youth-friendly treatment options.

### Aim

The aim of the study is to conduct a systematic review concerning the effectiveness of mental health treatments conducted within any YFHS, IYS or ‘one-stop shop’ clinics. The primary research question of the review is as follows:

- What effect do mental health interventions given at ‘youth-friendly’ clinics for treating mental health, such as IYS and YFHS, have on the mental health and quality of life (QoL) of young people?

## Method

The systematic review will adhere to guidelines set out in the Preferred Reporting Items for Systematic reviews and Meta-Analyses (PRISMA) 2020 statement.^30^,^32^ The research process was initiated in the beginning of 2024 with preliminary searches for similar reviews in the field in the PubMed, Cochrane Library and PROSPERO databases. Searches yielded no similar systematic reviews on the subject. Hence, a comprehensive research strategy was formulated. A protocol for study procedure was published in the PROSPERO database (ID nr CRD42024528687) in May of 2024.

### Patient and public involvement statement

None.

### Identifying relevant studies

In order to ensure high quality of included data in the systematic review, only studies published in peer-reviewed journals will be eligible for inclusion. Hence, no grey literature will be included in the systematic review. The Population, Intervention, Control and Outcome (PICO) statement for the review is as follows: the targeted population is people aged between 12 and 25 years of age. This age range is a common definition of the patient group for YFHS and IYS,^27 33^ which also largely corresponds to the WHO definition of ‘youth’.^34^ To maximise inclusivity, any study where a majority, >50%, of the study population falls within this age range will be included.

The interventions should be classified as ‘youth-friendly’. Any studies focusing on services labelled ‘youth-friendly’ or ‘integrated youth services’ offering treatment for mental health concerns will be accepted in the screening process. No further specification concerning treatment interventions, for example psychotherapy using cognitive behavioural therapy, nor treatment given by any specific professional category, such as general physicians or psychologists, will be applied. The inclusion criteria for studies have been purposefully designed to be wide in order to capture a range of interventions, from early intervention to care for severely ill patients. Treatment of specific diagnoses will also be included in the review, given that services are ‘youth-friendly’. However, studies evaluating treatments for other conditions than mental health, such as physical health or sexual and reproductive health, will be excluded. Studies focusing on ‘community services’ that are not specifically YFHS or IYS will not be included in the present review, partially to limit the scope of the review but also since reviews partially covering this topic already exist.^29 35^

Due to a current lack of common terminology in the research field, studies describing ‘youth-friendly’ health services often use varying terms to describe these services. Although the relevant services are often called ‘youth-friendly’ or ‘youth-centred’ in literature, the usage of these terms is not universal. To identify relevant studies that described ‘youth-friendly’ services for the systematic review described in the present protocol, two criteria were used in both title and abstract screening and full-text screening of studies for inclusion in the systematic review.

1. Studies using the terms ‘youth-friendly’, ‘youth-centred’, YFHS, IYS, ‘one-stop shop’ or similar to describe their services were included in the study.
2. Studies describing interventions that corresponded to the WHO standards for YFHS or that corresponded to descriptions of known IYS, such as headspace,^36^ were included in the study.

Apart from these criteria, whether articles referenced known studies describing YFHS or IYS was also used as a secondary criterion when selecting studies for full text review.

In order to identify all relevant studies for the present review, any studies with comparisons between groups (controlled) or with a baseline (prepost) will be included in the review. Examples of study designs that will be included are randomised controlled trials (RCT) and pragmatic RCT as well as observational and case-control studies. Single cross-sectional data collections, case series, single case reports and expert opinion studies will be excluded. In studies where control groups are used, these must consist either of young people receiving mental healthcare interventions at ‘regular’ or ‘traditional’ healthcare clinics, treatment at other services or in a waiting list protocol for inclusion.

Finally, to be included in the review, studies must use outcome measures of either mental health (eg, Patient Health Questionnaire-9 (PHQ-9) or Becks Depression Inventory (BDI) for depression and Generalized Anxiety Disorder 7-item scale (GAD-7) for anxiety), general psychological functioning (eg, Clinical Outcomes in Routine Evaluation (CORE)) or QoL (eg, EQ-5D or Short Form Health Survey (SF-36)). To limit the scope of the review to a cohesive whole, no studies focusing only on patient satisfaction or general functioning will be included in the review. Also, while YFHS and IYS both have holistic approaches to health,^17 26^ the focus of the proposed review is primarily clinical, which entails that measures of ‘wellness’ or ‘well-being’^37^ will not be specifically sought in the review, although measures of psychological functioning and QoL may partially capture an increased well-being in patients. Only studies published in 2012 or later will be included in the study. This date was chosen as the guidelines regarding YFHS from the WHO were published in 2012. Only studies in English or Swedish will be included in the review.

### Search strategy

The search strategy was developed in collaboration with a search strategy specialist at Uppsala University Library and reviewed by another university librarian using the PRESS Peer Review Guidelines.^38^ In order to conduct the search, trial searches on different blocks in the search strategy were conducted in PubMed. The following search blocks, which in themselves contain numerous search terms, were formulated: “Mental health”, “Young people”, “Health care”, “Treatment outcome” and “Youth-friendly”. When the search was performed, the search blocks “Mental health”, “Young people”, “Health care” and “Treatment outcome” were combined to identify articles that had all of these search blocks included as the subject matter of the article (as defined by, eg, Medical Subject Headings (MeSH) terms or by phrases included in the title/abstract). The “Youth-friendly” search block was included in its entirety to identify all relevant studies on this theme. A final pilot search of the entire search algorithm was carried out in PubMed. The search algorithm can be found in online supplemental appendix 1. The search was also adapted from PubMed to additional databases: PsycINFO, Web of Science and CINAHL. The full literature search was performed on the 29th of May 2024. The search result yielded a total of 12 738 references. The titles and abstracts of these articles were screened between June 2024 and January 2025.

The result of the title and abstract screening was discussed at a seminar including experts in the field on the 10th of January 2025. During this discussion, it was found that the search algorithm did not adequately capture certain IYS services, particularly in Canada. This led to further trial searches in PubMed, also including the search terms “one stop shop”, after which it was decided that the planned follow-up search would include a separate search block consisting of the words ‘integrated’ and ‘youth’ within three words from each other in title/abstract. Results from this search block were then filtered with the ‘mental health’ and ‘young people’ search blocks. The final follow-up search, including this new search block, was carried out on the 29th of April 2025, yielding a further 2182 references. The search algorithm for this search can also be found in online supplemental appendix 1. Final screening of all references collected in the searches is ongoing, having started in May 2025, and is expected to be completed during 2025.

### Study selection

Title and abstract screening in the initial search were conducted using the online software Rayyan.^39^ Two reviewers, independently and blinded from each other, screened all titles and abstracts and marked studies to be included or to be excluded. At the end of the screening process, the review was opened up for comparison of results. Any conflicts between reviewers were resolved by input from a third member of the research group.

All studies chosen for inclusion in the review at this stage were reviewed in full text. Two reviewers again independently reviewed the articles and came to a consensus on inclusion through discussion. A third reviewer was available for consensus discussions in the event of disagreement. At this point, the reference lists of all articles selected in the screening process were also searched for additional studies that may fulfil the inclusion criteria, which in turn were handled in a similar way as the papers included in the first step. This process is currently being repeated for the follow-up search carried out in April 2025.

All coauthors of the study have been involved in reaching a consensus on the search strategy as well as which data to be extracted and on the final list of articles to be reviewed in full length.

### Data extraction and risk of bias assessment

Once study selection has been completed, the first author (MA) will extract information from the included studies using a standardised, preprepared template set up in Windows Excel. The template will be piloted on two studies chosen for inclusion to be able to adjust the template to the field of research and the type of studies found. After extraction, another member of the research group will validate the results extracted, and a third member will be called in to resolve any disagreements that have come up during data extraction. See table 1 for information on what kind of data will be extracted.

**Table 1:** Data to be extracted from studies identified in the search

Study design and descriptive information	For example, reference, authors, location and trial type
Participants	For example, sex, age, diagnosis and ethnicity
Intervention	For example, setting, type of mental health intervention and dose
Comparisons (when applicable)	For example, type, setting and dose
Outcomes	Mental health outcomes, QoL measures, etc.
Effects measures	Any reported
Other relevant information	

A risk of bias assessment of the included studies will be done. All studies will be assessed using the appropriate Cochrane risk of bias tools, depending on the type of study being assessed, and assessments will be done at study level.^40^ Characteristics assessed for bias include confounders, methods of randomisation (where applicable), drop-out, classification of participants, measurements and reporting of findings. The assessment of bias will be independently carried out by two researchers. Any conflicts in assessment will be resolved by consensus discussions with a third research group member. The GRADE approach will be used to assess the quality of the evidence at the outcome level for the end-of-intervention time point.^41^

### Data synthesis and analyses

The research group expects that the review will result in a selection of studies exploring the outcome of treatments at YFHS and IYS. Different strategies for synthesising data will be used depending on study characteristics. If sufficient studies using similar outcomes are found and are deemed to have acceptable heterogeneity, a meta-analysis of findings using a random effects model will be performed. Any outcome data to be included in the meta-analyses will be converted into appropriate effect measures, for example, standardised mean difference or OR. Heterogeneity will be determined using either chi^2^ or I^2^ depending on the number of studies per outcome measure. If 10 or more studies are found with a similar outcome, a funnel plot will be constructed in order to be able to assess any publication biases or selective reporting. If both randomised and non-randomised studies are identified in the search, separate meta-analyses will be performed. No sub-group or sensitivity analyses are planned in the present study, nor any meta-regression analyses. If a meta-analysis cannot be performed, a synthesis without meta-analysis will be performed, using guidelines for the presentation of studies.^42^

## Ethics and dissemination

The present study does not require any ethical approval since it consists of a review of previously published and ethically approved studies. However, the authors will ensure that all findings are accurately and ethically presented and adhere to ethical guidelines concerning research ethics in general.

The findings in the review will be disseminated through submission to open-access peer-reviewed journals. Abstracts of the results of the study will also be submitted to international scientific conferences within the field, and the results will also be disseminated in a local context, through local government and national networks for YFHS.

## Supplementary material

10.1136/bmjopen-2024-095714 online supplemental file 1
