# Supplementary material for: Effects of mental health interventions given at youth-friendly health services and integrated youth services: a systematic review protocol
Source: BMJ Open. 2025 Sep 21;15(9):e095714. doi: 10.1136/bmjopen-2024-095714 (PMC12458864; doi:10.1136/bmjopen-2024-095714)
Supplement: online supplemental file 1 [file bmjopen-15-9-s001.docx]

# APPENDIX 1: PUBMED SEARCH STRATEGY, Primary search and follow-up search

| Search no. 1  2024-05-29 | Search terms | Results |
| --- | --- | --- |
| 1 | "Mental Health Services"[Mesh] OR "Mental Health"[Mesh] OR "Mental Disorders"[Mesh] OR "Self-Injurious Behavior"[Mesh] OR "Substance-Related Disorders"[Mesh] | 1,667,754 |
| 2 | anorexi* OR anxi* OR "binge eating" OR bulimi* OR depress* OR "eating disorder*" OR melancholi* OR "mental disorder*" OR "mental disease*" OR "mental health" OR "mental illness*" OR "mental problem*" OR "mental state*" OR "mentally ill" OR neuros* OR neurotic OR psych* OR distress* OR emotion*  [Title/Abstract] | 2,194,712 |
| 3 | #1 OR #2 | 3,128,282 |
| 4 | "Adolescent"[Mesh] OR "Young Adult"[Mesh] OR "Adolescent Health Services"[Mesh] OR "Adolescent Medicine"[Mesh] | 2,727,105 |
| 5 | adolescen* OR boy OR boys OR girl* OR juvenile* OR pediatric* OR paediatric* OR teen* OR "young adult*" OR "young man" OR "young men" OR "young person*" OR "young people*" OR "young woman" OR "young women" OR youth*  [Title/Abstract] | 1,353,456 |
| 6 | #4 OR #5 | 3,476,129 |
| 7 | "Adolescent Health Services"[MeSH] | 5,923 |
| 8 | "adolescent care" OR clinic OR clinics OR facilit* OR "health care" OR healthcare OR "health cent*" OR "health service*" OR "primary care" OR provider* OR "psychiatric care" OR "psychiatric service*" OR "youth service*"  [Title/Abstract] | 2,313,813 |
| 9 | #7 OR #8 | 2,316,646 |
| 10 | "Treatment Outcome"[Mesh:NoExp] OR "Outcome Assessment, Health Care"[Mesh:NoExp] OR "Patient Health Questionnaire"[Mesh] | 1,266,235 |
| 11 | "clinical outcome*" OR "treatment outcome*" OR "treatment response*" OR "depression scale*" OR "anxiety scale*" OR "anxiety score*" OR "depression score*" OR "stress scale*" OR "patient reported outcome*" OR "mental health outcome*" OR "therapy outcome*" OR "therapeutic outcome*"  [Title/Abstract] | 495,642 |
| 12 | #10 OR #11 | 1,632,791 |
| 13 | #3 AND #6 AND #9 AND #12 | 11,717 |
| 14 | "adolescent friendl*" OR "adolescent responsive" OR "teenager friendl*" OR "teen friendl*" OR "youth friendl*" OR "youths friendl*" OR "youth centred" OR "youth oriented"  [Title/Abstract] | 1,240 |
| 15 | #13 OR #14 | 12,942 |
| 16 | #15 AND 2012:3000[pdat] | 9,059 |

| **Search no 2.**  **2025-04-29** | **Search terms** | **Results** |
| --- | --- | --- |
| 1 | "Mental Health Services"[Mesh] OR "Mental Health"[Mesh] OR "Mental Disorders"[Mesh] OR "Self-Injurious Behavior"[Mesh] OR "Substance-Related Disorders"[Mesh] | 1,727,942 |
| 2 | anorexi*[tiab] OR anxi*[tiab] OR "binge eating"[tiab] OR bulimi*[tiab] OR depress*[tiab] OR "eating disorder*"[tiab] OR melancholi*[tiab] OR "mental disorder*"[tiab] OR "mental disease*"[tiab] OR "mental health"[tiab] OR "mental illness*"[tiab] OR "mental problem*"[tiab] OR "mental state*"[tiab] OR "mentally ill"[tiab] OR neuros*[tiab] OR neurotic[tiab] OR psych*[tiab] OR distress*[tiab] OR emotion*[tiab] | 2,321,882 |
| 3 | #1 OR #2 | 3,284,284 |
| 4 | "Adolescent"[Mesh] OR "Young Adult"[Mesh] OR "Adolescent Health Services"[Mesh] OR "Adolescent Medicine"[Mesh] | 2,847,099 |
| 5 | adolescen*[tiab] OR boy[tiab] OR boys[tiab] OR girl*[tiab] OR juvenile*[tiab] OR pediatric*[tiab] OR paediatric*[tiab] OR teen*[tiab] OR "young adult*"[tiab] OR "young man"[tiab] OR "young men"[tiab] OR "young person*"[tiab] OR "young people*"[tiab] OR "young woman"[tiab] OR "young women"[tiab] OR youth*[tiab] | 1,430,904 |
| 6 | #4 OR #5 | 3,640,530 |
| 7 | "Adolescent Health Services"[MeSH] | 6,028 |
| 8 | "adolescent care"[tiab] OR clinic[tiab] OR clinics[tiab] OR facilit*[tiab] OR "health care"[tiab] OR healthcare[tiab] OR "health cent*"[tiab] OR "health service*"[tiab] OR "primary care"[tiab] OR provider*[tiab] OR "psychiatric care"[tiab] OR "psychiatric service*"[tiab] OR "youth service*"[tiab] | 2,501,789 |
| 9 | #7 OR #8 | 2,504,627 |
| 10 | "Treatment Outcome"[Mesh:NoExp] OR "Outcome Assessment, Health Care"[Mesh:NoExp] OR "Patient Health Questionnaire"[Mesh] | 1,322,207 |
| 11 | "clinical outcome*"[tiab] OR "treatment outcome*"[tiab] OR "treatment response*"[tiab] OR "depression scale*"[tiab] OR "anxiety scale*"[tiab] OR "anxiety score*"[tiab] OR "depression score*"[tiab] OR "stress scale*"[tiab] OR "patient reported outcome*"[tiab] OR "mental health outcome*"[tiab] OR "therapy outcome*"[tiab] OR "therapeutic outcome*"[tiab] | 546,195 |
| 12 | #10 OR #11 | 1,728,809 |
| 13 | #3 AND #6 AND #9 AND #12 | 12,736 |
| 14 | "adolescent friendl*"[tiab] OR "adolescent responsive"[tiab] OR "teenager friendl*"[tiab] OR "teen friendl*"[tiab] OR "youth friendl*"[tiab] OR "youths friendl*"[tiab] OR "youth centred"[tiab] OR "youth oriented"[tiab] | 1,373 |
| 15 | "integrated youth"[tiab:~3] AND #3 AND #9 | 118 |
| 16 | #13 OR #14 OR #15 | 14,179 |
| 17 | #16 AND 2012:3000[pdat] | 10,296 |
