# Supplementary material for: Effects of mental health interventions given at youth-friendly health services and integrated youth services: a systematic review protocol
Source: BMJ Open. 2025 Sep 21;15(9):e095714. doi: 10.1136/bmjopen-2024-095714 (PMC12458864; doi:10.1136/bmjopen-2024-095714)
Supplement: Supplementary file 2 [file bmjopen-2024-095714.reviewer_comments.pdf]

PEER REVIEW HISTORY

BMJ Open publishes all reviews undertaken for accepted manuscripts. Reviewers are asked to complete a checklist review form and are provided with free text boxes to elaborate on their assessment. These free text comments are reproduced below.

ARTICLE DETAILS

Title (Provisional)

Effects of mental health interventions given at Youth-Friendly Health Services and Integrated Youth Services: a systematic review protocol

Authors

Andersén, Mikael; Lostelius, Petra Vikman; Bring, Annika; Ring, Lena; Nystrand, Camilla; Olsson, Erik MG

VERSION 1 - REVIEW

**Reviewer**	**1**
**Name**	**Lutgens, Danyael**
**Affiliation**	**Capilano University**
**Date**	**09-Jun-2025**
**COI**	**None**

Re: Effects of mental health interventions given at Youth- Friendly Health Services and Integrated Youth Services: a systematic review protocol

Towards: BMJ

June 7, 2025

Overall comment:

High quality, comprehensive, systematic review proposed in a critical area of youth mental health. SR proposed has excellent screening and outcome measures.

Questions:

- What about specialized intervention services for specific diagnoses that are aligned with youth mental health – these are holistic but may not be included within the scope of this paper (ex. Early intervention)? Further definition of the types of services that will be included would be helpful here.

- Definition of youth services that includes ages 12 – 25 may be either to open on the one hand (12 years old) and too limited on the other (25 years old) – it seems your search criteria will be flexible enough, especially to capture newer services but confirm.

- Wondering about including such a large range of studies? Please provide rationale for including such variation in methodology (why not just RCTs?)

- Some references may be missing – for example, in the Canadian context, Malla et al., 2018.

- Really important to further clarify context here – studies from the US show rates of suicide attempts among young people rising, at the same time that there are funding cuts to social services. What is at stake here (which means that the type of studies being included is even more important)?

- Further clarify difference between “wellness” and “mental illness” as outcomes – ex: https://pmc.ncbi.nlm.nih.gov/articles/PMC7492887/ but there is more recent literature in this area

---

**Reviewer**	**2**
**Name**	**Boakye, Dorothy**
**Affiliation**	**University of Education Winneba, Health Administration and Education**
**Date**	**26-Jun-2025**
**COI**	**None**

This systematic review protocol addresses an important and timely research question regarding the effectiveness of youth-friendly mental health services. The topic is highly relevant given the increasing mental health challenges among young people and their reluctance to engage with traditional services. However, the manuscript requires significant revision across multiple sections before it meets the standards for publication in BMJ Open.

1. The abstract lacks a clear statement of the specific research question/hypothesis.

2. Include the PROSPERO registration number

Intoduction

3. The distinction between YFHS and IYS could be clearer earlier in the introduction

4. Consider adding a brief definition of "youth-friendly" services upfront

5. The knowledge gap could be stated more definitively

6. Line 94-95: "IYS have been established in the Western world" - this phrasing is somewhat vague and potentially exclusionary

Methods

Search strategy

7. The search appears to have been conducted in May 2024, but the protocol states data analysis will occur in "latter half of 2024" - this timeline seems inconsistent with a protocol paper

8. No mention of grey literature searching

9. No contact with experts in the field planned

10. The strategy appears incomplete and may compromise the comprehensiveness and precision of the search

Missing some key mental health conditions relevant to youth (e.g., ADHD, autism spectrum disorders, conduct disorders)

Could include "psychological wellbeing" or "emotional wellbeing"

Missing "self-harm" as a free-text term (only MeSH "Self-Injurious Behavior" included)

Consider adding "behavioral problems" or "behavioral disorders"

The free-text terms may be too broad (e.g., "boy" and "girl" without age qualifiers could capture studies on very young children).

Limited coverage of integrated services terminology

Missing community-based service terms

No mention of digital or online services

Lacks terms specific to mental health services settings

Critical Missing Terms:

"integrated service*" OR "community based" OR "community service*" OR "outreach service*" OR "digital health" OR "online service*" OR "telehealth" OR "one stop shop*" OR "drop in" OR "walk in" OR "peer support" OR "community mental health"

Outcomes related to Quality of life measures, Functional outcomes, Service utilization outcomes and Patient satisfaction measures missing.

Suggested Completion:

OR "quality of life" OR "functional outcome*" OR "psychosocial functioning" OR "social functioning" OR "academic performance" OR "school attendance" OR "service satisfaction" OR "patient satisfaction" OR "help seeking" OR "engagement" OR "retention"

11. Its unclear how many articles were retrieved after combining all search terms using the Boolean operator, language and date limits.

12. Pilot test the strategy to assess precision and recall

13. Document any protocol deviations if strategy is modified post-registration

Inclusion/Exclusion Criteria:

14. The definition of "youth-friendly" is circular and imprecise

15. Age range justification (12-25 years) needs stronger rationale

16. The >50% population criterion is problematic and could introduce bias

Discussion

17. The discussion doesn't adequately discuss anticipated challenges

18. There is limited discussion of how findings will inform practice, policy and research

19. Missing discussion of potential limitations in interpretation

---

VERSION 1 - AUTHOR RESPONSE

Reviewer: 1 (see also attached)
Dr. Danyael Lutgens, Capilano University
Comments to the Author:
Re: Effects of mental health interventions given at Youth- Friendly Health Services and Integrated Youth Services: a systematic review protocol
Towards: BMJ
June 7, 2025

Overall comment:

High quality, comprehensive, systematic review proposed in a critical area of youth mental health. SR proposed has excellent screening and outcome measures.

Questions:

<u>- What about specialized intervention services for specific diagnoses that are aligned with youth mental health – these are holistic but may not be included within the scope of this paper (ex. Early intervention)? Further definition of the types of services that will be included would be helpful here.</u>

Response: Thank you for your comment. We have attempted to further clarify that the scope for interventions is deliberately wide to ensure that the review captures a wide range of articles. See lines 153-156 and 158-160 in the revised manuscript for specific changes.

<u>- Definition of youth services that includes ages 12 – 25 may be either to open on the</u>

one hand (12 years old) and too limited on the other (25 years old) – it seems your search criteria will be flexible enough, especially to capture newer services but confirm.

Response: Thank you for your comment. A brief justification of the age range chosen is found on lines 127-128 in the original manuscript. However, a further justification of the age range chosen has been added on lines 145-146 in the revised manuscript. A deliberately wide age range was chosen in order not to miss relevant studies. Studies not relevant for the review will be excluded during screening.

- Wondering about including such a large range of studies? Please provide rationale for including such variation in methodology (why not just RCTs?)

Response: Thank you for your comment. The decision to include all study designs including pre-post measurements was made out of concern that few RCT studies may be available in the field, which was also a finding from trial searches early in the research process, and because other study designs other than RCTs may still provide useful data within the field. Hence, a broad scope of included studies was chosen to maximize the number of studies included in the review. A clarification of this rationale has been added on line 174 in the revised manuscript.

- Some references may be missing – for example, in the Canadian context, Malla et al., 2018.

Response: Thank you for your comment. It was discovered following screening of articles and in a seminar, that studies describing Canadian services were indeed missing form the search. This is now described on lines 208-210. After conducting further trial searches, a separate search block using "integrated" and "youth" within 3 words from each other in title/abstract was added in the follow-up search. This update to the search strategy is described on lines 212-214 in the revised manuscript.

- Really important to further clarify context here – studies from the US show rates of suicide attempts among young people rising, at the same time that there are funding cuts to social services. What is at stake here (which means that the type of studies being included is even more important)?

Response: Thank you for your comment. We agree that it's important to make clear what services are to be described in the proposed review. The scope of the review has been purposely broad to include both early intervention services as well as services focusing on severely ill patients, and a clarification concerning this may be found on lines 153-156 in the revised manuscript. We have also in the introduction endeavoured to be clear concerning what's at stake, and this has been further elaborated on in lines 76-78 in the revised manuscript.

- Further clarify difference between “wellness” and “mental illness” as outcomes – ex: https://pmc.ncbi.nlm.nih.gov/articles/PMC7492887/ but there is more recent literature in this area

Response: Thank you for your comment. A clarification concerning “wellness” and “mental illness” in the review has been added on lines 184-188.

Reviewer: 2
Ms. Dorothy Boakye, University of Education Winneba
Comments to the Author:
This systematic review protocol addresses an important and timely research question regarding the effectiveness of youth-friendly mental health services. The topic is highly relevant given the increasing mental health challenges among young people and their reluctance to engage with traditional services. However, the manuscript requires significant revision across multiple sections before it meets the standards for publication in BMJ Open.

Abstract
1. The abstract lacks a clear statement of the specific research question/hypothesis.
Response: Thank you for your comment. This has now been added on line 33-35.

2. Include the PROSPERO registration number
Response: Thank you for your comment. The PROSPERO registration ID number can be found on line 55 as well as line 119-120 in the original manuscript, lines 61 and 133-134 in the revised manuscript.

Introduction
3. The distinction between YFHS and IYS could be clearer earlier in the introduction
Response: Thank you for your comment. The descriptions of YFHS and IYS is in the second paragraph of the introduction (starting on line 87 and 97 respectively. We think that it is important to frame the development of these services with the status of mental health of the young people utilizing the clinics, a description of which is to be found in the first paragraph of the introduction. We have now added a further discussion of the distinction between YFHS and IYS, line 95-97 and line 105-106 and 109-110 in the revised manuscript.

4. Consider adding a brief definition of "youth-friendly" services upfront
Response: Thank you for your comment. A further definition of the “youth-friendly” guidelines have been added on lines 95-97 in the revised manuscript.

5. The knowledge gap could be stated more definitively

Response: Thank you for your comment. We have further described the knowledge gap in the revised manuscript, with additions on the subject being found on lines 114-115 and 117-118.

6. Line 94-95: "IYS have been established in the Western world" - this phrasing is somewhat vague and potentially exclusionary.

Response: Thank you for your comment. We agree that this wording is unfortunate, and changes have been made in the manuscript, to be found on line 104 in the revised manuscript. A similar wording has also been corrected in the Abstract, line 30.

Methods

Search strategy

7. The search appears to have been conducted in May 2024, but the protocol states data analysis will occur in "latter half of 2024" - this timeline seems inconsistent with a protocol paper

Response: Thank you for your comment. We agree that it would have been preferable to have published the present protocol paper prior to the commencement of the systematic review described in the protocol. Unfortunately, due to time constraints within the project, the protocol describes an ongoing project and hence work on the systematic review described in the protocol is in progress, which is reflected in the protocol. At the time of writing, the analysis of data for the systematic review has not yet started, why we still feel that the protocol study is highly relevant for publication. The timeline in the protocol has been updated to reflect progress in the project since submission of the protocol study for peer review, and updates can be found in the revised manuscript on lines 41-42, 47-49 and 208-219.

8. No mention of grey literature searching

Response: Thank you for your comment. To limit the scope of the proposed systematic review, and to ensure a uniformly high quality of included data, no grey literature will be included in the systematic review. A clarification to this effect has been added on lines 141-143 in the revised manuscript.

9. No contact with experts in the field planned

Response: Thank you for your comment. In the initial protocol, no contact with experts in the field was planned. However, after the submission of the present protocol study the study procedure was amended to include contact with experts in the field. A seminar to discuss an ongoing PhD project, which includes the study protocol, was

carried out on the 10th of January 2025. A description of this seminar and outcomes there-of can be found on lines 208-216.

10. The strategy appears incomplete and may compromise the comprehensiveness and precision of the search
Missing some key mental health conditions relevant to youth (e.g., ADHD, autism spectrum disorders, conduct disorders)
Could include "psychological wellbeing" or "emotional wellbeing"
Missing "self-harm" as a free-text term (only MeSH "Self-Injurious Behavior" included)
Consider adding "behavioral problems" or "behavioral disorders"
The free-text terms may be too broad (e.g., "boy" and "girl" without age qualifiers could capture studies on very young children).
Limited coverage of integrated services terminology
Missing community-based service terms
No mention of digital or online services
Lacks terms specific to mental health services settings

Critical Missing Terms:
"integrated service*" OR "community based" OR "community service*" OR "outreach service*" OR "digital health" OR "online service*" OR "telehealth" OR "one stop shop*" OR "drop in" OR "walk in" OR "peer support" OR "community mental health"
Outcomes related to Quality of life measures, Functional outcomes, Service utilization outcomes and Patient satisfaction measures missing.

Suggested Completion:
OR "quality of life" OR "functional outcome*" OR "psychosocial functioning" OR "social functioning" OR "academic performance" OR "school attendance" OR "service satisfaction" OR "patient satisfaction" OR "help seeking" OR "engagement" OR "retention"

Response: Thank you for your comprehensive comment. Constructing an exhaustive search strategy is a challenge, and we have wetted several of your suggestions during trial searches, but they were found to yield few if any new references. Search terms concerning wellbeing were discussed during trials, and while we agree that this concept has relevance to the topic for the review, trial searches showed that the search terms “psych* OR emotion*” at least partially covered the same references, while inclusion of the terms risked leading to additional references not relevant to the subject of the review being found. Discussion concerning wellbeing as an outcome has been added on lines 184-188.

The present study protocol focuses specifically on mental health and changes in mental health. While studies including quality of life and different measures of functioning may

be included in the review, we have in the searches focused on measures of mental health, and not functioning or patient satisfaction. A comment clarifying this has been added on line 183-184.

After a seminar including experts in the field (see point 9 above), it was decided to expand the search criteria in anticipation of the planned follow-up search, since coverage in the search of IYS was insufficient. In the follow-up search, a separate search block using “integrated” and “youth” within 3 words from each other in title/abstract was added, since this solution yielded promising results in trial searches. In the trial searches, different options, including adding “one stop shop” as a search term was considered, but was found to yield no relevant further references. This update to the search strategy is described on lines 210-214 in the revised manuscript and the search strategy in the follow-up search may be found in Appendix 1.

Partially to limit the scope of the present review, and partially because other review articles at least partially cover the same topic, it was decided to focus the present review on on-site services and not to include studies specifically covering online service and telehealth. The same reasoning was applied to community services, where review articles are already available. In trial searches as well as in the searches conducted as part of the review, it was also found that the searches conducted also partially covered the field of community services, and that studies including Settipani et. al., 2019, as well as Savaglio, O’Donnell, Hatzikiriakidis, Vicary and Skouteris, 2022, were screened as part of the searches. We believe that community services aimed at youth and executed in a youth-friendly fashion are adequately covered for by the scope of this systematic review without the addition of specific search terms to cover these services. A clarification concerning the scope of the review with certain justification for choices has been added in the revised manuscript on line 158-160.

11. Its unclear how many articles were retrieved after combining all search terms using the Boolean operator, language and date limits.

Resonse: Thank you for your comment. The number of articles retrieved in the different steps of the searches can be found in Appendix 1. The appendix has also been updated to also cover findings from the follow-up search carried out in April 2025 (see comments above).

12. Pilot test the strategy to assess precision and recall

Resonse: Thank you for your comment. In the original manuscript, “trial searches” in PubMed are mentioned on line 152, but we agree that this passage is unclear. Information concerning the final pilot test of the search strategy has been added and can be found on line 201-202 in the revised manuscript.

13. Document any protocol deviations if strategy is modified post-registration
Resonse: Thank you for your comments. In response to above comments, the protocol study has been updated to reflect deviations and updates to the protocol made after the submission of the protocol study for review. These changes have also been updated in the protocol submitted to the PROSPERO database.

Inclusion/Exclusion Criteria:
14. The definition of "youth-friendly" is circular and imprecise

Resonse: Thank you for your comment. We agree that defining “youth-friendly” services for the present protocol has been a difficult task, as no commonly agreed terminology exists for “youth-friendly” interventions, as discussed in the original manuscript lines 220-230. In the revised manuscript, we have added a paragraph describing considerations in selecting studies describing “youth-friendly” services, to be found on lines 161-173.

15. Age range justification (12-25 years) needs stronger rationale

Resonse: Thank you for your comment. A further justification of the age range chosen has been added on lines 144-146 in the revised manuscript.

16. The >50% population criterion is problematic and could introduce bias

Resonse: Thank you for your comment. As stated on lines 129-130 in the original manuscript, the decision to include articles where >50% of the population is within the age range was made to maximize the numbers of articles found for screening purposes. Any exacerbated risk of bias concerning what studies are found in the search will be ameliorated in the study selection process, and that the goal to maximize studies for screening takes precedence.

Discussion
17. The discussion doesn't adequately discuss anticipated challenges

Resonse: Thank you for your comment. At the request of the editors, the discussion section has been removed entirely, as a Discussion section is not required in a protocol study.

18. There is limited discussion of how findings will inform practice, policy and research

Resonse: Thank you for your comment. At the request of the editors, the discussion section has been removed entirely, as a Discussion section is not required in a protocol study.

19. Missing discussion of potential limitations in interpretation

Resonse: Thank you for your comment. At the request of the editors, the discussion section has been removed entirely, as a Discussion section is not required in a protocol study.
